# Understanding the Knowledge, Perceptions, and Attitudes of University Students Toward Blood Donation and the Turkish Red Crescent: A Cross-Sectional Study

**DOI:** 10.7759/cureus.68216

**Published:** 2024-08-30

**Authors:** Mikail Batu, İlknur Aydoğdu Karaaslan, Gül Bülbül Maraş, Burcu Öksüz, Şehriban Kayacan, İlkay Burak Taşkıran

**Affiliations:** 1 Department of Public Relations and Advertising, Necmettin Erbakan University, Konya, TUR; 2 Department of Journalism, Ege University, İzmir, TUR; 3 Vocational School of Health Service, İzmir Democracy University, İzmir, TUR; 4 Department of Public Relations and Advertising, İzmir Kâtip Çelebi University, İzmir, TUR; 5 Corporate Communications, CeyBer Educational Technologies, İzmir, TUR; 6 Department of Public Relations and Advertising, İstanbul Yeni Yüzyıl University, İstanbul, TUR

**Keywords:** attitudes, knowledge, university students, non-governmental organization, perception, turkish red crescent, blood donation

## Abstract

Background

The need for blood arising from illness, surgery, and accidents is met through donations. With global population growth, both non-governmental organizations and public institutions are vital for blood supply and distribution. The Turkish Red Crescent, a leading non-governmental organization in blood donation and humanitarian aid, excels in both national and international contexts, ensuring a safe blood supply and reinforcing social solidarity during crises. This study aims to determine the knowledge, perceptions, and attitudes of students from different departments at a public university in Turkey regarding blood donation and the Turkish Red Crescent.

Methodology

This was a cross-sectional study. The study population included 34,442 Ege University students, with the sample consisting of 385 students who continued their education online due to the February 6, 2023, earthquake in Turkey and voluntarily participated in the study. Data were collected via an online survey.

Results

The study found that 56.9% (219) of the participants were female, 30.9% (119) were in the second year, and 16.6% (64) were from the Faculty of Science. Overall, 89.6% (345) of the students held positive views on blood donation. Further, 64.4% (248) showed a positive inclination toward donating blood, and 76.8% (296) viewed it as a social responsibility. However, 70.4% (271) of the students had doubts about whether the blood will reach those in need, and 61.6% (237) were concerned about this issue. Additionally, women donated blood more than men with significant differences in some attitudes based on gender.

Conclusions

The research revealed that students defined blood donation as a good deed and an indicator of human solidarity. On the other hand, students lacked knowledge about the Turkish Red Crescent and were undecided in some of their opinions about the Turkish Red Crescent.

## Introduction

Civil society organizations can be defined as “entities operating independently from governmental agencies, lobbying efforts to achieve political, economic, socio-cultural, legal, and similar objectives, engaging in persuasion and actions, attracting its members and employees on a voluntary basis and non-profit organizations that also secure their revenues through donations and memberships” [1]. Besides positioning themselves in the civil sphere, civil society organizations also maintain relationships with the government and the private sector. The readiness of societal structures for unforeseen events is of vital importance. The activities of individuals acting voluntarily meet various needs in many areas of social life [2].

One of the most significant civil society organizations that has evolved throughout history in Turkey is the Turkish Red Crescent (Türk Kızılayı). “The Red Crescent is an open-system organization that operates in a collective relationship with the society it serves and recruits members at the national level, as well as with international organizations to which it is affiliated at the international level” [3].

The Turkish Red Crescent, which is seen as one of the best organizations in terms of disaster management, performs work within the scope of “international aid, migration and refugee services, social services, health, first aid, education, youth and mineral water enterprises,” with its priority on blood needs and disaster situations [4]. It provides emergency response services on issues such as “earthquake, house fire, flood, excessive snowfall, dam gate explosion, bridge collapse, collapse or mining accident, landslide, tornado/storm, terrorism” [5].

Among the goals of the Turkish Red Crescent is to provide services abroad when needed and deliver necessary aid to foreign countries [6]. Turkish Red Crescent Blood Services is the most important blood-providing institution in Turkey and meets half of the blood needs of the country [7].

Despite extensive research on blood donation worldwide, studies on this topic in Turkey remain limited. Yıldız et al. [8] investigated the reasons for the population’s reluctance to donate blood in a southern city of Turkey. Kaya et al. [9] examined the influence of university students’ attitudes and knowledge on blood donation. Çelik and Güven [10] aimed to develop a valid scale to measure these attitudes. Özgür et al. [11] highlighted the difficulty in finding blood donors, attributing it primarily to the public’s misconceptions, beliefs, attitudes, and behaviors regarding blood donation. University students, due to their dynamic nature and awareness of blood donation, are considered capable of meeting a significant proportion of the blood supply. Therefore, this study aims to reveal the perceptions and attitudes of university students about the Turkish Red Crescent and blood donation. In this context, a study was conducted among Ege University students. University students, particularly in terms of civil society organizations, are of great importance to the Red Crescent. The strong and continuous relationships between the Turkish Red Crescent and university students play a critical role not only in terms of students donating blood but also in increasing solidarity throughout society by getting support from students.

Objectives

The primary objective of this study was to reveal public university students’ knowledge, perceptions, and attitudes toward the Turkish Red Crescent and blood donation. The secondary objective was to determine how these knowledge, perceptions, and attitudes vary according to students’ demographic characteristics and other factors. It also sought to identify which faculty at the public university had students who were more willing to donate blood. The following study hypotheses were formulated:

H1: The knowledge, perceptions, and attitudes of students toward the Red Crescent vary according to their demographic characteristics (gender, class, faculty).

H2: The knowledge, perceptions, and attitudes of students toward blood donation vary according to their demographic characteristics (gender, class, faculty).

H3: The students’ following of the Red Crescent on social media influences their perceptions and attitudes toward blood donation.

H4: The students’ thoughts about the Red Crescent’s blood donation centers influence their perceptions and attitudes toward blood donation.

H5: The awareness of students about the Red Crescent’s blood donation campaigns influences their perceptions and attitudes toward blood donation.

H6: The students’ knowledge of where the Red Crescent’s blood donations are used influences their perceptions and attitudes toward blood donation.

H7: The ease of reaching the Red Crescent when students want to donate blood influences their perceptions and attitudes toward blood donation.

H8: The students’ awareness of the Red Crescent blood donation points in Izmir influences their perceptions and attitudes toward blood donation.

## Materials and methods

Study setting and sample

This was a cross-sectional study. The study population consisted of 34,442 students enrolled at Ege University in western Turkey. The sample comprised students who voluntarily participated in the study and were continuing their education online due to the earthquake that occurred in Turkey on February 6, 2023. A convenience sampling method was employed, allowing the researchers to select participants based on their availability and willingness to participate. The sample size of the study was evaluated based on the sample table provided by Yazıcı and Eroğlu (2004) [12], indicating that the minimum required sample size at a 0.05 significance level for a population size of 10,000 was 381. The study population consisted of 385 university students. This study was conducted through the collaboration of researchers from different fields of expertise. During the course of the study, four researchers were affiliated with Ege University, one with İzmir Katip Çelebi University, and one with İzmir Democracy University. The researchers were able to collaborate effectively due to their presence in the same city.

Inclusion criteria

The inclusion criteria were as follows: participants needed to be currently enrolled students at Ege University, at least 18 years of age for informed consent, willing to voluntarily agree to participate in the study, and have access to the online survey platform.

Exclusion criteria

The exclusion criteria were as follows: students enrolled in universities other than Ege University, participants under the age of 18, individuals who were not enrolled at Ege University (e.g., faculty, staff, alumni), those who did not voluntarily agree to participate in the study, and surveys with incomplete responses were excluded.

Data collection tool

Data were collected using the Students Identification Form, Knowledge and Perceptions of the Turkish Red Crescent Form, and Blood Donation Attitude Scale. The Students Identification Form and Knowledge and Perceptions of the Turkish Red Crescent Form developed by researchers in line with the comprehensive literature consists of 30 questions such as age, gender, grade level, faculty, and monthly income status [1,3-6,10]. Çelik and Güven’s (2015) Blood Donation Attitude Scale was used in the study [10]. The Blood Donation Attitude Scale is a 24-item, five-point Likert-type scale consisting of three sub-dimensions that measure attitudes toward blood donation. The lower dimensions include social responsibility, concern, and social opinion and understanding. In all three dimensions, question items are scored as 1 (strongly disagree), 2 (disagree), 3 (undecided), 4 (agree), and 5 (strongly agree). It was developed by Çelik and Güven (2015) and underwent validity and reliability assessments [10]. In addition, a reliability test was conducted for the study, and Cronbach’s alpha value was found to be 0.69. As Cronbach’s alpha value was between 0.60 and 0.80, it was very reliable [12].

Ethical considerations

Ethics committee approval was obtained by the Scientific Research and Publication Ethics Committees of the Rectorate of Ege University with the decision number 04/05 dated 06. 04. 2023 and the protocol number 1860. This study was supported by the Izmir Red Crescent Regional Directorate. Consent was obtained or waived by all study participants. The participants were given a URL to access the survey online survey and were asked on the first page if they agreed to complete the questionnaire. The participants were directed to a linked page containing important study information before completing the questionnaire. The completion of the survey form took approximately 12-15 minutes.

Statistical analysis

This study is descriptive. Alongside descriptive statistics, frequency distributions, chi-square analysis, t-test to compare means, and one-way analysis of variance analyses were interpreted in the evaluation of the data. Multiple comparison tests were also applied when necessary. When differences between groups emerged, post hoc tests were used to reveal which group caused the difference. While analyzing and interpreting the data, they were tested at a significance level of 0.05. The test performed when p < 0.05 at the end of the analysis was considered statistically significant [12].

## Results

Students’ perceptions and attitudes toward the Red Crescent and blood donation by sociodemographic characteristics

It was determined that 56.9% (219) of the students participating in the study were female and 43.1% (166) were male. The blood donation attitudes of the students were evaluated according to the demographic characteristics of gender (t = 0.532; p = 0.595), class (F = 0.422; p = 0.864), and faculty (Table 1).

**Table 1:** Comparison of students’ demographic characteristics with the Blood Donation Attitude Scale score means. X̅ ± SD = total score mean ± standard deviation; - = not available.

Demographic characteristics				Blood donation means			
		N	%	X	SD	Test value	P-value
Gender	Female	219	56.9	81.68	7.50	t = 0.532	0.595
	Male	166	43.1	81.19	10.25		
Grade	Preparatory	8	2.1	80.00	7.15	F = 0.422	0.864
	1st Grade	108	28.1	82.21	7.07		
	2nd Grade	119	30.9	80.61	9.75		
	3rd Grade	53	13.8	81.73	7.85		
	4th Grade	70	18.2	81.51	8.24		
	5th Grade	22	5.7	82.50	12.98		
	6th Grade	5	1.3	80.40	16.37		
Faculty	Faculty of Literature	31	8.1	80.77	6.62	F = 1.253	0.217
	Engineering Faculty	30	7.8	80.36	8.36		
	Faculty of Science	64	16.6	80.31	9.71		
	Faculty of Economics and Administrative Sciences	30	7.8	83.43	6.03		
	Faculty of Agriculture	26	6.8	79.69	10.11		
	Faculty of Medicine	4	1.0	84.25	15.96		
	Faculty of Education	21	5.5	86.09	10.32		
	Faculty of Communication	42	10.9	82.33	7.39		
	Faculty of Nursing	7	1.8	77.57	10.53		
	Faculty of Sport Sciences	4	1.0	78.50	4.43		
	Faculty of Health Sciences	13	3.4	82.07	5.48		
	Faculty of Dentistry	16	4.2	79.87	6.23		
	Faculty of Pharmacy	49	12.7	81.95	9.19		
	Çeşme Faculty of Tourism	1	0.3	97.00	-		
	Faculty of Fine Arts Design and Architecture	3	0.8	77.33	6.65		
	Birgivi Faculty of Islamic Sciences	2	0.5	87.50	0.70		
	Ödemiş Faculty of Health Sciences	2	0.5	73.00	4.24		
	Faculty of Law	2	0.5	90.50	4.94		
	Vocational School of Higher Education	38	9.9	81.18	10.43		

Students’ perceptions and attitudes toward blood donation

As shown in Table 2, 89.6% (345) of the students were positive about blood donation, and 91.4% (352) thought that donating blood was religiously appropriate. This shows that there is no religious prejudice among the relevant masses while collecting blood for the Red Crescent and creates a positive situation for the Red Crescent.

**Table 2:** Distribution of students’ perceptions and attitudes toward blood donation.

Scale items	Strongly disagree (%)	Disagree (%)	Undecided (%)	Agree (%)	Strongly agree (%)
Giving blood is a sign of human solidarity	3.4 (13)	2.3 (9)	4.7 (18 )	46.2 (178)	43.4 (167)
It is not religiously appropriate to give blood	71.7 (276)	19.7 (76)	7.8 (30)	0.5 (2)	0.3 (1)
A person who gives blood feels peaceful	2.3 (9)	4.4 (17)	28.8 (111)	44.9 (173)	19.5 (75)
Giving blood without expecting anything in return is a sign of goodness	1.0 (4)	1.3 (5)	4.4 (17)	42.9 (165)	50.4 (194)
Giving blood is a source of happiness	2.9 (11)	9.4 (36)	30.4 (117)	36.6 (141)	20.8 (80)
I feel the happiness of fulfilling some social duties after donating blood	2.9 (11)	4.4 (17)	15.8 (61)	50.6 (195)	26.2 (101)
Donating blood enhances social commitment.	1.6(6)	8.6(33)	14.0(54)	46.8(180)	29.1(112)
The blood I donate can save someone’s life	1.0 (4)	0.3 (1)	3.1 (12)	32.7 (126)	62.9 (242)
Donating blood strengthens the sense of social responsibility	1.0 (4)	3.4 (13)	7.8 (30)	48.6 (187)	39.2 (151)
After the donation, the staff’s caring behaviors influence me positively	1.8 (7)	3.6 (14)	12.5 (48)	43.9 (169)	38.2 (147)
I consider blood donation as a beneficial act for humanity	1.3 (5)	0.5 (2)	4.7 (18)	53.2 (205)	40.3 (155)
I believe it is a social responsibility that every individual should do	1.6 (6)	14.0 (54)	15.3 (59)	40.5 (156)	28.6 (110)
I am hesitant about giving blood because I am not sure that the blood I give reaches those in need	5.5 (21)	11.2 (43)	13.0 (50)	20.5 (79)	49.9 (192)
I think that the institution to which I donate blood does not deliver the blood to those in need	6.8 (26)	11.9 (46)	19.7 (76)	24.2 (93)	37.4 (144)
The length of the pre-donation questionnaire makes me give up on giving blood	17.7 (68)	43.9 (169)	23.1 (89)	8.1 (31)	7.3 (28)
Waiting before giving blood makes me give up	20.5 (79)	42.3 (163)	19.2 (74)	12.2 (47)	5.7 (22)
The physical discomfort of a previous donor (difficulty in finding a vein, excessive bleeding, etc.) can impact my decision to donate blood	16.9 (65)	26.5 (102)	15.1 (58)	29.1 (112)	12.5 (48)
I don’t donate blood due to the fear of experiencing a physical decline in my body before the donation	24.9 (96)	38.7 (149)	15.3 (59)	16.9 (65)	4.2 (16)
The hygiene of the staff, facility, and medical equipment affects my idea of giving blood	1.8 (7)	2.3 (9)	3.6 (14)	44.9 (173)	47.3 (182)
The dexterity of the nurse who will take blood affects my idea of the next blood donation	3.9 (15)	11.2 (43)	13.2 (51)	31.2 (120)	40.5 (156)
I donate blood because it is considered a good deed	36.6 (141)	26.8 (103)	19.2 (74)	12.5 (48)	4.9 (19)
I donate blood to get someone’s blessing.	38.2 (147)	22.1 (85)	16.1 (62)	17.4 (67)	6.2 (24)
Since men are stronger than women, they should donate more blood	45.2 (174)	28.1 (108)	16.1 (62)	6.8 (26)	3.9 (15)
If I do not have any health insurance, I donate blood to know my state of health and to have a blood test	26.2 (101)	23.4 (90)	23.4 (90)	21.3 (82)	5.7 (22)

The majority of participants (64.4%, n = 248) had a positive attitude toward blood donation. A high percentage, 93.3% (n = 359), believed that blood donation is an act of goodness, while 51.2% (n = 197) viewed it as a source of happiness. Additionally, 76.8% (n = 296) viewed it as a social responsibility, and 75.9% (n=292) believed it enhances social bonds. A notable 95.6% (n = 368) thought that blood donation saves lives, and 86.8% (n=334) felt it strengthens social responsibility.

Moreover, 82.1% (n = 316) of participants reported that positive staff behavior post-donation improved their intention to donate again. A vast majority of participants, specifically 93.5% (n = 360), believed that blood donation is beneficial to humanity. However, 70.4% (n = 271) were concerned about whether their blood reaches those in need, and 61.6% (n = 237) doubted it, highlighting the need for better informational efforts. Problems during the donation process affected 41.6% (n = 160) of participants’ willingness to donate. The importance of cleanliness in the donation process was emphasized by 92.2% (n = 355) of the participants, and 71.7% (n = 276) said that a nurse’s skill impacts their decision to donate.

Regarding gender differences, analysis (F = 1.253; p = 0.217) showed no significant difference in overall blood donation motivations, although specific scale items revealed that men donated more frequently than women (p < 0.001). Men donated more regularly than women across various intervals, with 83.3% of men donating every two years compared to 56.3% annually. Reasons for not donating included hemophobia and health concerns, particularly affecting women.

There was a significant difference between the gender of the students and the intervals at which they donated blood (p < 0.001). Men donated more blood than women. The proportion of men who donated blood once every two years was 83.3% (10), the rate of men who donated blood once every six months was 73.1% (19), the rate of men who donated blood at intervals of more than two years was 70.7% (29), and the rate of men who donated blood once a year was 56.3% (27). There was a significant relationship between the genders of students who did not donate blood and the reasons that deterred them from blood donation. In particular, 22.2% (48) of women stated that they did not donate blood due to various diseases, 17.1% (37) due to hemophobia and fear of needles, and 36.6% (79) due to reasons affecting blood donation such as weight and age. In addition to these reasons, they did not donate blood due to their fear of disease transmission (1.4%, 3) and religious reasons (0.5%, 1). Overall, 84.1% (37) of the students could not donate blood due to hemophobia and fear of needles, and 71.6% (48) of women did not donate blood due to various diseases. There was a significant relationship between the gender of the students participating in the study and their attitudes of “Giving blood strengthens the sense of social responsibility” (p = 0.038). In particular, 62.3% (94) of women strongly agreed with this statement, while this rate was 37.7% (57) in men. While 56.7% (106) of women stated that they agreed with this statement, the same expression rate was 43.3% (81) in men. While 61.5% (8) of male students stated that they strongly disagreed, this rate was 38.5% (5) for girls. While the rate of undecided men was 53.3% (16), the rate of women was 46.7% (14). It is also noteworthy that 100% of those who answered strongly disagree by gender were male students.

There was a significant relationship between the responses of the students participating in the study to the statement “I am hesitant about donating blood because I am not sure that the blood I give reaches those in need” according to their gender (p = 0.041). While 76.7% (33) of women stated that they did not agree with this statement, this rate was 23.3% (10) in men. As 62.0% (31) of women and 38% (19) of men stated that they were undecisive, 57% (45) of women and 43% (34) of men agreed, 52.1% (100) of women and 47.9% (92) of men strongly agreed, there was a significant difference between the hesitation of the blood given by the students to reach the needy according to gender.

According to the gender of the students, there was a significant difference for the Red Crescent regarding the statement “I think that the institution to which I donate blood does not deliver the blood to those in need” (p = 0.015). While 51.4% (74) of men strongly agreed with this attitude, this rate was 48.6% (70) for women. While 54.8% (51) of women stated that they agreed, 45.2% (42) of men also stated that they agreed. While 54.8% (51) of women expressed that they agreed, the rate was 45.2% (42) for men. Overall, 76.1% (35) of women and 23.9% (11) of men did not agree with this statement, while 65.4% (17) of women and 34.6% (9) of men strongly disagreed.

The attitudes toward “The length of the questionnaire applied before donation causes me to give up donating blood” varied according to the genders of the students participating in the study (p = 0.001). Men declared that they generally agreed with this attitude. While the rate of men who said they definitely agreed was 71.4% (20), this rate was 28.6% (8) for women. While the rate of men who agreed with this statement was 58.1% (18), the rate of women was 41.9% (13). Overall, 53.9% (48) of those who stated they were indecisive were women and 46.1% (41) were men.

There was a relationship at the 0.05 significance level among the responses given by students based on their genders to the statement “Waiting before donating blood causes me not to give up” (p = 0.029). Overall, 57% (45) of women strongly disagreed compared to 43% (34) of men. Additionally, while 63.8% (104) of women did not agree with this statement, 36.2% (59) of men also expressed that they did not agree. In cases of indecision, the rate of women was slightly higher than men at 55.4% (41). While 53.2% (25) of men agreed that waiting before giving blood discouraged them, this rate was 46.8% (22) for women. Meanwhile, 68.2% (15) of men completely agreed with this statement, and only 31.8% (7) of women stated that they agreed with this statement.

According to the gender of the students participating in the study, there was a significant relationship between the statements “The hygiene of the staff, the place and the medical items affect my blood donation idea” (p = 0.026). While 85.7% (6) of men strongly disagreed with this attitude, this rate was 14.3% (1) for women. On the other hand, 63.7% (116) of women strongly agreed with this statement, while the rate of men was 36.3% (66). In addition, 52.6% (91) of women agreed with this statement, and 47.4% (82) of men stated that the hygiene of Red Crescent employees, space, and medical items affected their thoughts about donating blood.

The answers people gave to the statement “I donate blood because giving blood is a good deed” also differed at the level of 0.05% significance between the genders of the students participating in the study (p = 0.011). While 68.4% (13) of men strongly agreed with this statement, this rate was 31.6% (6) for women. The rate of agreement of men and women with this statement was equal at 50% (24). While 67% (69) of women disagreed with this statement, this rate was 33% (73) for men. While 51.8% (73) of women strongly disagreed, this rate was 48.2% (68) of men. When their indecision status was evaluated, 63.5% (47) of women stated that they were indecisive, while this rate was 36.5% (27) for men. Women thought that when they give blood, it is not a religious duty, but a humanitarian responsibility.

There was a relationship between the gender of the students and their answers to the attitude “I donate blood to receive someone’s blessing” at the 0.05 significance level (p = 0.029). While 50.3% (74) of women strongly disagreed with this statement, this rate was 49.7% (73) for men. In addition, while 67.1% (57) of women disagreed with this statement, this rate was 32.9% (28) of men. Overall, 61.2% (41) of women agreed with this statement, and 38.8% (26) of men also agreed. On the other hand, 62.5% (15) of men strongly agreed with this statement, while this rate was 37.5% (9) for women. While the rate of undecisive women was 61.3% (38), 38.7% (24) of men stated that they were indecisive.

There was a significant relationship (p < 0.001) between the attitudes of students participating in the study and their perception of the activities of the Turkish Red Crescent (Kızılay). Overall, 75% (6) of females and 25% (2) of males found it very successful, while 76.4% (42) of females and 23.6% (13) of males found it successful. Additionally, 58.5% (83) of males and 41.5% (59) of females perceived Kızılay as very unsuccessful.

There was a relationship at the 0.05 significance level (p = 0.039) between students’ genders and their opinions about the Turkish Red Crescent’s website. Overall, 56.9% (37) of males and 43.1% (28) of females considered it very bad, while 51.9% (27) of males and 48.1% (25) of females thought it was bad. Moreover, 66.7% (30) of females believed it was good, while only 33.3% (15) of males shared this view. The ratio of both females and males considering it very good was equal at 50% (1). Females also exhibited a higher level of indecision (61.1%, 135) compared to males (38.9%, 86).

There was a significant relationship between students’ genders and their opinions about the Turkish Red Crescent (Kızılay) (p = 0.001). While 55.6% (79) of males perceived Kızılay as a very bad institution, this percentage was 44.4% (63) for females. In contrast, 81.8% (9) of females expressed that it is very good, while only 18.2% (2) of males shared this sentiment. Among females, 66.7% (52) exhibited an undecided attitude, whereas this rate was 33.3% (26) among males. Females showed a higher tendency toward indecision in their opinions.

There was a significant relationship between students’ genders and their belief in the possibility of donating blood to another organization other than the Turkish Red Crescent (Kızılay) (p = 0.014). While 63.3% (31) of females and 36.7% (18) of males believed that blood donation could not be made to another institution, 50.2% (106) of females and 49.8% (105) of males thought that blood donation could be made to another place outside of Kızılay.

There was a significant relationship between students’ genders and the times they donated blood to the Turkish Red Crescent (Kızılay) (p < 0.001). Notably, men donated more blood compared to women. Within the last one year, 60.2% (53) of males and 39.8% (35) of females had donated blood; within the last two years, 67.6% (23) of males and 32.4% (11) of females had donated blood; within the last five years, 81% (17) of males and 19% (4) of females had donated blood; and within the last 10 years, 70% (7) of males and 30% (3) of females had donated blood. On the contrary, 71.6% (166) of females and 28.4% (66) of males stated that they had never donated blood.

There is a significant relationship between students’ genders and their preferred blood donation locations on the Ege University campus at the 0.05 significance level (p = 0.03). Among female students, 66.7% (46) preferred Mediko, while 33.3% (23) of males did. For the Faculty of Dentistry, 55% (22) of females and 45% (18) of males preferred it. In terms of campus dormitories, 86.7%(13) of females and 13.3% (2) of males showed a preference. Additionally, around the Faculty of Economics and Administrative Sciences, 71.4% (15) of females and 28.6% (6) of males preferred blood donation locations. Interestingly, 60% (3) of males and 40% (2) of females preferred locations near the Department of Textile Engineering. In addition to the differentiation, of note, both male and female students preferred the areas near the library equally with a rate of 50% (88).

Students’ perceptions and attitudes toward Red Crescent and blood donation according to their faculties

The participation rates in the study varied across different faculties and schools. The Faculty of Science had the highest participation at 16.6% (64), followed by the Faculty of Pharmacy at 12.7% (49), the Faculty of Communication at 10.9% (42), Vocational School at 9.9% (38), the Faculty of Arts at 8.1% (31), and the Faculties of Engineering and Economics and Administrative Sciences sharing the same percentage of 7.8% (30). Additionally, the Faculty of Agriculture had a participation rate of 6.8% (26), the Faculty of Education of 5.5% (21), the Faculty of Dentistry of 4.2% (16), and the Faculty of Health Sciences of 3.4% (13).

There was a significant relationship at the 0.05 significance level (p = 0.004) among the reasons motivating students to donate blood based on their faculties. Students in the Faculty of Science (15.9%, 11) and the Faculty of Engineering (13%, 9) believed that donating blood would be beneficial for their health. Students from the faculties of Education, Communication, Engineering, and Sports Sciences expressed that they donated blood to benefit others, without considering personal gain, at an equal rate of 16.7% (5). Students from the faculties of Literature, Communication, Science, and Economics and Administrative Sciences also shared the same rate (11.1%, 4) in expressing motivation based on empathy. All students from the Vocational School (100%, 2) stated that they donated blood for religious reasons. Students from the faculties of Science, Education, and Pharmacy equally (20%, 4) mentioned a sense of personal responsibility as their motivation. Additionally, 20% (4) of students from the faculties of Science and Communication stated that they donated blood due to a sense of social responsibility. All students from the Faculty of Science (100%, 2) declared that they donated blood due to some past painful experiences.

When the reasons for not donating blood according to students’ faculties were evaluated, a significant relationship was observed (p < 0.001). There was a significant relationship between the faculties of the students and their responses to the attitude that giving blood is an indicator of human solidarity (p < 0.001). Overall, 11.2% (20) of students in the Faculty of Pharmacy and Communication and 19.2% (32) of students in the Faculty of Science strongly agreed with this statement. Moreover, 23.1% (3) of the students in the Faculty of Economics and Administrative Sciences and 15.4% (2) of the students studying in Sports Sciences strongly disagreed with this statement.

According to the faculties, there was a significant relationship between the answers of the students to the statement “I am happy to fulfill some of my social duties after giving blood” (p = 0.015). Students in the Faculty of Science (15.9%, 31), Pharmacy (13.8%, 27), Communication (13.3%, 26), and Vocational School (12.8%, 25) stated that they fully agreed with this attitude. On the other hand, students of the Faculty of Science (36.4%, 4), Engineering (18.2%, 2), and Vocational School (18.2%, 2) declared that they did not completely agree with this idea. Of note, students differed in their levels of agreement, indicating a growing indifference toward societal responsibilities and possibly highlighting the prominence of individualism.

There was a significant relationship (p = 0.006) at the 0.05 significance level between students’ perception of blood donation as a beneficial act for humanity and their respective faculties. Students from the Faculty of Science (18.7%, 29), Faculty of Pharmacy (14.2%, 22), Faculty of Economics and Administrative Sciences (12.3%, 19), and Faculty of Communication (10.3%, 16) strongly agreed with the view that blood donation is a beneficial act for humanity. The rates in other faculties were observed to be below 10%(9). However, of note, despite the strong agreement, there were certain percentages of students in the Faculty of Science (40%, 2), Vocational School (40%, 2), and Ödemiş Health Sciences Faculty (20%, 1) who disagreed with this perspective.

There was a significant relationship (p = 0.002) between the faculties students attended and their levels of familiarity with the Turkish Red Crescent (Kızılay). Students from the faculties of Literature, Science, and Pharmacy equally indicated that they did not know the Turkish Red Crescent, with a rate of 18.2% (2) in each. However, some students from the faculties of Science (22.4%, 17), Pharmacy (18.4%, 14), and Communication (10.5%, 8) expressed that they were very well acquainted with Kızılay.

The attitudes of students toward Kızılay’s blood donation centers varied according to their faculties (p < 0.001). Students from the faculties of Literature and Communication (21.3%, 10) and Engineering (14.9%, 7) perceived Kızılay’s blood donation centers as bad, while 34.2% (13) of students from the Faculty of Science, 13.2% (5) of Pharmacy and Vocational School students, and 10.5% (4) of Communication Faculty students considered them very bad. Despite this, 25% (4) of Pharmacy students, 12.5% (2) of students from the Birgivi Faculty of Islamic Sciences, 12.5% (2) of Vocational School students, 17.6% (25) of students from the Faculty of Science, 13.5% (19), of Pharmacy students, and 10.6% (15) of vocational school students believed that Kızılay’s blood donation centers are good.

The perceptions of students about Kızılay as an institution varied according to their faculties (p < 0.001). The opinions of students in the faculties of Engineering, Science, and Vocational Schools were the same, with a rate of 18.2% (12). Similarly, students in the faculties of Agriculture, Dentistry, Faculty of Islamic Sciences (Birgivi), and Ödemiş Health Sciences Faculty also shared the same rate of 9.1% (4). On the other hand, students in the faculties of Science, Communication, and Pharmacy thought that Kızılay was very bad, with rates of 19.7% (28), 14.8% (21), and 12% (17), respectively. Students in the faculties of Science, Engineering, Communication, and Pharmacy considered Kızılay as bad, with rates of 12.1% (11), 14.3% (13), 13.2% (12), and 11% (10), respectively.

According to the faculties, the points where the students went to give blood on the Ege University campus also varied (p < 0.001). Overall, 15.3% (27) of the students in the Faculty of Science, 10.8% (19) of the students in the Faculty of Engineering, 10.2% (18) of the students in the Faculty of Agriculture, 10.8% (18) of the students in the Faculty of Pharmacy, 11.4% (20) of the students in the Vocational School preferred areas around the Library. Overall, 29% (20) of the students in the Faculty of Science, 24.6% (17) of the students in the Faculty of Pharmacy around SKS (Medico); 32.5% (13) of the students in Communication, 25% (10) of Dentistry students around Dentistry; the students in the Faculty of Engineering, Economics and Administrative Sciences, Faculty of Medicine, Faculty of Pharmacy and Vocational School preferred the vicinity of Textile Engineering at an equal rate of 20% (1). Further, 20.3% (1) of the students in the Faculty of Medicine, 11.9% (7) of the students studying in the Faculty of Economics and Administrative Sciences and Pharmacy, and 13.6% (8) of the students in the Vocational School stated that they preferred areas around the Faculty of Medicine. The majority (71.4%, 15) of the Economics and Administrative Sciences region was preferred by students studying in that department. Students staying in the dormitory also indicated the points around the dormitory. This rate was 13.3% (2) of the students in the Faculty of Science, 20% (3) of the students in the Faculty of Communication and Pharmacy, and 26.7% (4) of the students in the Vocational School.

Students’ knowledge and perceptions of the Turkish Red Crescent and blood donation means

When the students’ knowledge about the Red Crescent and their blood donation means was evaluated, there was a difference between the students’ knowledge about blood donation campaigns and their blood donation means (t = -2.082; p = 0.038). There was no significant difference observed between students in terms of following the Turkish Red Crescent on social media (t = 0.998; p = 0.319), their opinions about the Turkish Red Crescent’s blood donation centers (F = 0.588; p = 0.672), their awareness of where Turkish Red Crescent blood donations are used (t = 0.54; p = 0.878), their ease of reaching Turkish Red Crescent when they wanted to donate blood (t = 0.934; p = 0.351), and their awareness of Turkish Red Crescent blood donation points in Izmir (t = -0.021; p = 0.983) along with blood donation means (Table 3).

**Table 3:** Comparison of students’ knowledge and perceptions about the Turkish Red Crescent and blood donation means. X̅ ± SD = total score mean ± standard deviation.

Statements	Students’ knowledge and perceptions about the Red Crescent			Means of blood donations			
		N	%	X	SD	Test value	P-value
Their status of following the Turkish Red Crescent on social media	Yes	35	9.1	82.88	10.95	t = 0.998	0.319
	No	350	90.9	81.33	8.54		
Their opinions about the Turkish Red Crescent’s blood donation centers	Very Bad	38	9.9	80.31	10.99	F = 0.588	0.672
	Bad	47	12.2	80.46	9.52		
	Undecisive	142	36.9	82.07	8.09		
	Good	142	36.9	81.35	8.61		
	Very Good	16	4.2	82.87	8.60		
Their awareness of the Turkish Red Crescent’s blood donation campaigns	Yes	289	75.1	80.93	9.10	t = -2.082	0.038
	No	96	24.9	83.08	7.56		
Their awareness of where Turkish Red Crescent blood donations are used	Yes	194	50.4	81.54	9.50	t = 0.154	0.878
	No	191	49.6	81.40	8.01		
Their ease of reaching the Turkish Red Crescent when wanting to donate blood	Yes	297	77.1	81.70	8.62	t = 0.934	0.351
	No	88	22.9	80.70	9.33		
Their awareness of Turkish Red Crescent blood donation points in Izmir	Yes	268	69.6	81.46	8.98	t = -0.021	0.983
	No	117	30.4	81.48	8.35		

Students’ demographic characteristics with blood donation attitude scale subscale score mean

The Blood Donation Attitude Scale consists of three sub-dimensions, which are social and societal responsibility, concern, and social views and understanding. When students’ attitudes toward blood donation were evaluated in the context of gender, a significant difference was observed between gender and social and societal responsibility, with females being more likely to donate blood due to social and societal responsibility feelings (t = 2.267; p = 0.024). No significant relationship was observed between gender and concern (t = -0.917; p = 0.360) and social views and understanding (t = -1.471; p = 0.142) dimensions. There was no significant difference observed among the classes students attend when evaluated in terms of dimensions. However, a significant difference was observed between students’ faculties in terms of social and societal responsibility dimensions (F = 1.856; p = 0.018) (Table 4).

**Table 4:** Comparison of students’ demographic characteristics with Blood Donation Attitude Scale subscale score means. X̅ ± SD = total score mean ± standard deviation; - = not available.

Demographic characteristics		Social and societal responsibility			Concern			Social views and understanding		
		X	SD	P-value	X	SD	P-value	X	SD	P-value
Gender	Female	46.92	6.05	0.024	25.90	5.14	0.360	8.84	3.22	0.142
	Male	45.42	6.89		26.40	5.52		9.36	3.65	
Grade	Preparatory	44.25	4.23	0.509	26.87	5.66	0.412	8.87	3.48	0.076
	1st Grade	47.25	5.85		25.63	5.17		9.31	3.00	
	2nd Grade	46.04	7.47		25.55	5.55		9.01	3.46	
	3rd Grade	46.01	6.71		27.03	4.66		8.67	2.99	
	4th Grade	46.07	5.20		27.04	4.81		8.40	3.32	
	5th Grade	45.59	6.41		26.13	7.02		10.7	5.07	
	6th Grade	42.80	9.52		26.40	6.18		11.20	5.26	
Faculty	Faculty of Literature	45.70	4.72	0.018	27.29	4.56	0.207	7.77	2.62	0.054
	Faculty of Engineering	44.76	7.27		26.43	6.12		9.16	2.87	
	Faculty of Science	46.00	7.01		25.78	5.33		8.53	3.20	
	Faculty of Economics and Administrative Sciences	47.23	5.66		27.66	4.78		8.53	2.66	
	Faculty of Agriculture	43.69	7.03		26.26	6.10		9.73	3.79	
	Faculty of Medicine	45.75	5.56		27.50	6.02		11.00	6.21	
	Faculty of Education	50.52	5.36		25.90	5.36		9.66	4.30	
	Faculty of Communication	47.16	6.16		27.21	4.34		7.95	2.63	
	Faculty of Nursing	44.85	7.12		23.28	4.75		9.42	2.82	
	Faculty of Sport Sciences	44.75	4.27		22.75	5.12		11.00	1.63	
	Faculty of Health Sciences	48.61	4.95		22.53	5.75		10.92	3.52	
	Faculty of Dentistry	44.06	5.88		27.37	3.48		8.43	3.46	
	Faculty of Pharmacy	46.59	5.61		25.77	5.79		9.59	3.97	
	Çeşme Faculty of Tourism	56.00	-		32.00	-		9.00	-	
	Faculty of Fine Arts Design and Architecture	48.66	10.21		21.66	6.65		7.00	3.00	
	Birgivi Faculty of Islamic Sciences	47.50	7.77		28.00	11.31		12.00	2.82	
	Ödemiş Faculty of Health Sciences	36.00	4.24		27.50	0.70		9.50	0.70	
	Faculty of Law	54.50	2.12		26.00	2.82		10.00	4.24	
	Vocational School	45.92	7.39		25.02	5.19		10.23	3.82	

Students’ knowledge and perception of the Red Crescent and blood donation attitude scale sub-dimension score mean

When evaluating students’ knowledge about the Turkish Red Crescent, a significant difference was observed between their thoughts about the Turkish Red Crescent’s blood donation centers and the concern dimension (F = 8.150; p = 0.000). Additionally, there was a significant difference among blood donors regarding the awareness of where their blood donations are utilized, influencing their decision to donate blood (t = -0.20; p = 0.028). Students stated that they did not donate blood because they did not know where their blood donations were used. There is a significant relationship between students’ ability to easily reach the Red Crescent when they want to donate blood and their blood donation (t = -2.70; p = 0.007). The difficulty in reaching the Turkish Red Crescent to donate blood affected their decision to donate blood (Table 5).

**Table 5:** Comparison of students’ knowledge status about the Red Crescent and Blood Donation Attitude Scale sub-dimension score means. X̅ ± SD = total score mean ± standard deviation.

Students’ knowledge status about the Red Crescent		Social and societal responsibility			Concern			Social views and understanding		
		X	SD	P-value	X	SD	P-value	X	SD	P-value
The students’ status of following the Turkish Red Crescent on social media	Yes	47.14	6.88	0.408	26.37	6.02	0.773	9.37	3.96	0.582
	No	46.19	6.42		26.10	5.23		9.03	3.36	
Their opinions about the Turkish Red Crescent’s blood donation centers	Very bad	43.55	9.26	0.000	28.89	5.48	0.000	7.86	3.45	0.111
	Bad	44.57	6.57		27.06	5.40		8.82	3.44	
	Undecisive	45.90	5.83		26.85	4.65		9.30	3.31	
	Good	47.57	5.75		24.68	5.16		9.09	3.35	
	Very good	49.56	6.01		23.06	6.80		10.25	4.26	
Their awareness of the Turkish Red Crescent’s blood donation campaigns	Yes	46.05	6.71	0.236	25.98	5.45	0.363	8.89	3.44	0.095
	No	46.95	5.61		26.55	4.83		9.57	3.29	
Their awareness of where Turkish Red Crescent blood donations are used	Yes	46.73	6.36	0.167	25.53	5.63	0.028	9.27	3.70	0.235
	No	45.82	6.54		26.72	4.89		8.85	3.09	
Their ease of reaching the Turkish Red Crescent when wanting to donate blood	Yes	46.78	5.97	0.005	25.73	5.35	0.007	9.18	3.50	0.215
	No	44.57	7.67		27.45	4.95		8.67	3.11	
Their awareness of Turkish Red Crescent blood donation points in Izmir	Yes	46.68	6.27	0.062	25.80	5.26	0.075	8.97	3.53	0.417
	No	45.35	6.80		26.85	5.36		9.28	3.14	

## Discussion

Studies have been conducted on attitudes and behaviors regarding blood donation in Turkey and various countries. In a study by Kaplan (2018), women have more positive attitudes toward blood donation, and men’s self-confidence in donating blood was found to be higher, resulting in a higher intention to donate blood [13]. Özpulat (2017) conducted a study on university students to determine their thoughts on blood and organ donation, finding that attitudes toward blood donation differ based on gender [14]. In the study by Efteli et al. (2018) on nursing students, the mean of the social responsibility sub-dimension was higher in female participants [15]. This study found a significant difference in blood donation between male and female students, with males donating more than females. The connection between gender and social responsibility was also significant, with females donating blood due to social responsibility feelings. Another significant finding from the study was that students with knowledge about blood donation had higher donation rates. While students generally have a positive attitude towards blood donation, providing more information could alleviate their doubts and encourage them to donate.

In the study conducted by Gader et al. (2011), people opposed importing blood from abroad and believed that blood donation was a religious obligation [16]. In the study by Kasraian and Maghsudlu (2012) in Iran, most donors made blood donations for altruistic reasons [17]. Zaller et al. (2005) conducted a study in China, revealing that motivating factors for blood donation include social pressure, the desire to know screening results, and altruism [18]. Harrington et al. (2007) conducted a study in Ireland, where donors cited being aware of patients’ needs (88%, 467), trusting the blood transfusion service (71%, 374), and a promotional campaign (70%, 368) as reasons to encourage blood donation [19]. In the study by Bart et al. (2014) study in Switzerland, hope for saving lives and solidarity emerged as the two most important reasons for donating blood [20]. Soriano (2019) conducted a study in the Philippines, where the primary reason for blood donation was familial medical needs [21]. In the study conducted by Cantürk et al. (2013) in Turkey, the most common reason for donation was determined as being in need of one of their relatives/acquaintances [22]. According to the findings obtained within the scope of this study, students stated that they donated blood for health, empathy, and personal and social responsibility. They stated that they donated blood for religious reasons, bad experiences, and not wanting to faint at the sight of blood. Unlike other studies, the most important factor for the participants in this study was the health benefits of blood donation. In a study by Tulunay (2007), the idea of saving lives was identified as the most important motivation for blood donation. Informing the community was found to be the most crucial factor in increasing blood donation, and the Turkish Red Crescent Blood Center was the most preferred institution for blood donation [23]. Similarly, in this study, the most preferred centers for blood donation were those affiliated with the Turkish Red Crescent.

Reasons for individuals who did not donate blood include fear, lack of time, weakness, and health reasons related to age [16]. Zaller et al. (2005) conducted a study in China, where inhibiting factors included fear of infection and other adverse health effects, including the fear of loss of life [18]. In the study by Harrington et al. (2007) conducted in Ireland, major reasons given by non-donors for not donating included medical reasons, lack of information, fear of needles, and time constraints [19]. In the study by Bart et al. (2014) in Switzerland, the two main obstacles to blood donation were found to be a lack of information about where and when to donate and the deferral of donation [20]. Soriano (2019) conducted a study in the Philippines, the most common considerations for not donating were health conditions and lack of resources [21]. In a study by Yıldız et al. (2006), the most significant obstacle to blood donation was determined to be people’s indifference and ignorance [8]. In a study by Altındiş (2019), participants expressed fears of infection during the blood donation process and concerns about the hygiene of the materials used [24]. Kocabaş and Eke (2021) found that the working hours of the Turkish Red Crescent were not suitable for blood donation and the perception that there were already too many blood types similar to theirs were the reasons for stopping blood donation [25]. In this study, various factors such as fear of contracting diseases, fear of needles, insufficient weight, prejudice, anemia, lack of knowledge about donation centers, distrust for institutions, and alcohol and drug use were mentioned as reasons for not donating.

The study by Bostancı-Daştan et al. (2013) investigated the blood donation rates of health college students in Turkey, revealing that the participants’ blood donation rates were above the national mean [26]. A majority of the students (61.5%) expressed a positive attitude toward future blood donation, although their knowledge about blood donation was found to be at a moderate level. In this study, no significant difference was observed between the students’ faculties and their general attitudes toward blood donations.

In the study, it was determined that students were indecisive about their general opinions regarding the Turkish Red Crescent. While some students evaluated the activities of the Turkish Red Crescent negatively, others assessed them positively. Most students claimed to be familiar with the Turkish Red Crescent, but there were noticeable results among those who knew little or did not know at all. It was also observed that some participants did not know the phone number of the Turkish Red Crescent call center, some were aware of the Turkish Red Crescent’s mobile application, knew about blood donation campaigns, and were informed about the activities of the Turkish Red Crescent, but were not aware of its social media accounts. In this context, participants seemed to lack sufficient information about the communication channels of the Turkish Red Crescent. University students play a crucial role in increasing the activity and effectiveness of civil society organizations in the community. The more active students are in civil society organizations, the more progress these organizations will make [27], and students will get to know these institutions better. In this study, it can be said that university students in Turkey should take a more active role in civil society organizations.

Limitations and strengths

This study has several limitations and strengths. One significant limitation is the possibility of bias in self-reported data from students, which may not fully represent the general public’s views on the Turkish Red Crescent and blood donation. Additionally, the study’s reliance on online surveys due to the earthquake may limit the generalizability of the findings to students who were not affected or were unable to participate. On the other hand, the study’s strengths include a large sample of university students, which provides valuable insights into their views and understanding of blood donation and the Turkish Red Crescent. The findings highlight significant gaps in awareness and knowledge, which should guide focused communication initiatives to improve student involvement and blood donation rates.

## Conclusions

It was determined that factors such as pre-blood donation practices and waiting periods in Turkey, insecurity and hesitations regarding the delivery of donated blood to those in need, and concerns about the negative impact of blood donation on health reduce blood donation motivation. Free blood tests, the idea of ​​saving lives, a sense of social/personal responsibility, and empathy were determined to be important elements in encouraging blood donation. It was determined that the Red Crescent, which coordinates blood donations at the national level in Turkey, is highly recognized and aware of its activities; however, its work has largely been unsuccessful. In this context, it may be considered that the Red Crescent carries out activities aimed at its corporate image. In Turkey, large-scale studies are needed with different populations regarding blood donation and the perception of the Red Crescent.

## Appendices

Understanding University students’ knowledge, perceptions and attitudes towards blood donation and the Turkish Red Crescent: A cross-sectional study

We invite you to participate in the study titled “Understanding University Students’ Knowledge, Perceptions and Attitudes Toward Blood Donation and the Turkish Red Crescent: A Cross-Sectional Study,” conducted by Assoc. Prof. Dr. Mikail Batu. Before deciding to participate, it is important to understand the purpose and methods of the research. Please read and understand this form carefully. If you have any questions or need further information, feel free to ask. Participation in this study is entirely voluntary. You may choose to withdraw from the study at any time after starting. Your response will be considered as consent to participate. Please answer the questions on the forms without any external pressure or suggestions. All personal information will be kept confidential and used solely for research purposes.

If you agree to participate voluntarily, please select the appropriate option below:

a. Yes, I agree to participate in the study voluntarily.

b. No, I do not agree to participate in the study voluntarily.

Dear students,

Data for this study will be collected using three tools: the “Students Identification Form,” “Knowledge and Perception of the Turkish Red Crescent Form,” and the “Blood Donation Attitude Scale.” Completing the survey will take approximately 12-15 minutes. In the scale, please choose the option that best reflects your opinion for each statement. Your responses will be used solely for academic purposes and will be handled with the utmost ethical considerations. Thank you for your participation and interest. If you wish to proceed, please start answering the questions.

Students Identification Form

1. Gender

a. Woman         b. Man

2. Age groups

a. 18-20 year     b. 21-23 year     c. 24-26 year     d. 26 years and above

3. What is your body weight?

a. 40-49 kg    b. 50-59 kg    c. 60-69 kg     d. 70 kg and above

4. Which year are you in at university?

a. Preparation   b. Grade 1    c. Grade 2     d. Grade 3      e. Grade 4      e. Grade 5   f. Grade 6

5. Which faculty are you studying in?

a. Faculty of Literature

b. Faculty of Engineering

c. Faculty of Science

d. Faculty of Economics and Administrative Sciences

e. Faculty of Agriculture

f. Faculty of Medicine

g. Faculty of Education

h. Faculty of Communication

i. Faculty of Nursing

j. Faculty of Sport Sciences

k. Faculty of Health Sciences

l. Faculty of Dentistry

m. Faculty of Pharmacy

n. Cesme Tourism Faculty

o. Faculty of Fisheries

p. Faculty of Fine Arts, Design and Architecture

q. Birgivi Faculty of Islamic Sciences

r. Ödemiş Faculty of Health Sciences

s. Faculty of Law

t. Vocational School

6. Monthly family income

a. Less than 10.000 TL   b. 10.000-15.000 TL     c. 16.000-20.000 TL    d. More than 20.000 TL

7. Have you ever donated blood to the Turkish Red Crescent?

a. Yes             b. No

8. If you have donated blood to the Turkish Red Crescent before, how often do you donate blood to the Turkish Red Crescent?

a. At intervals of less than six months          b. Once every six months            c. Once a year

d. Once every two year                                    e. At intervals longer than two years

9. What motivates you to donate blood?

a. The idea that it will be beneficial for health

b. Altruism / Altruism

c. Empathy/Humility

d. Religious reasons

e. Sense of personal responsibility

f. Sense of social responsibility

g. Bitter experience

h. Optimizer

ı. Spousal, friend, and family support

10. If you have not donated blood to the Turkish Red Crescent before, what are the reasons that keep you from donating blood?

a. Religious reasons           b. Fear of disease               c. Bloodsickness /Fear of needles           d. Various diseases

Knowledge and Perception of the Turkish Red Crescent Form

11. How well do you know Turkish Red Crescent?

a. I don't know any         b. I know very little          c. I know          d. I know very well

12. Are you aware of the activities of the Turkish Red Crescent?

a. Yes               b. No

13. How successful do you consider the Turkish Red Crescent?

a. Very unsuccessful           b. Failed        c. Undecided          d. Successful          e. Very successful

14. Are you aware of the Turkish Red Crescent’s social media accounts?

a. Yes               b. No

15. Do you follow Turkish Red Crescent on social media?

a. Yes               b. No

16. What do you think about the Turkish Red Crescent’s website?

a. Very bad          b. Bad         c. Undecided    d. Good        e. Very good       

17. What do you think about the Turkish Red Crescent’s blood donation centers?

a. Very bad          b. Bad          c. Undecided          d. Good         e. Very good       

18. What do you think about the Turkish Red Crescent’s blood donation practices?

a. Very bad          b. Bad          c. Undecided        d. Good          d. Very good       

19. Which of the following describes your thoughts about the Turkish Red Crescent as an institution?

a. Very bad          b. Bad          c. Undecided        d. Good          d. Very good       

20. What do you think about the benefit of the Turkish Red Crescent’s humanitarian aid activities to those in need?

a. It’s no use           b. No benefit          c. Undecided               d. Useful           e. Very useful

21. Are you aware of the Turkish Red Crescent’s blood donation campaigns?

a. Yes               b. No

22. Can blood donations be made anywhere other than the Turkish Red Crescent?

a. Yes               b. No

23. Do you know the number of the Turkish Red Crescent call center?

a. Yes               b. No

24. Did you know that the Turkish Red Crescent has a mobile application?

a. Yes               b. No

25. Do you know where the Turkish Red Crescent uses blood donations?

a. Yes               b. No

26. Is the Turkish Red Crescent’s support important to increase blood donation in Turkey?

a. Yes               b. No

27. Do you have easy access to the Turkish Red Crescent when you want to give blood?

a. Yes               b. No

28. Do you know the Turkish Red Crescent blood donation points in Izmir?

a. Yes               b. No

29. When was the last time you gave blood to the Turkish Red Crescent?

a. I never gave         b. In the last 1 year     c. In the last 2 years       d. In the last 5years 

e. In the last 10 years

30. Which point on the Ege University campus would you prefer to give blood?

a. Near the library

b. Near SKS (Medico)

c. Near the Faculty of Dentistry

d. Around Textile Engineering

e. Around the dormitories on campus

f. Near the Faculty of Medicine,

g. Near the Faculty of Economics and Administrative Sciences

**Table 6:** Blood Donation Attitude Scale.

Items	Strongly disagree	Disagree	Undecided	I agree	Absolutely agree
1. Giving blood is a sign of human solidarity					
2. Giving blood is not religiously permissible					
3. People who give blood feel peaceful					
4. Giving blood without expecting anything in return is an act of kindness					
5. Giving blood is a source of happiness					
6. After giving blood, I feel happy to fulfill some of my social duties					
7. Giving blood increases social cohesion					
8. My blood can save someone’s life					
9. Giving blood strengthens a sense of social responsibility					
10. The relevant behavior of the staff after the donation positively influenced me effects					
11. I see blood donation as a good deed for humanity					
12. I think it is a social responsibility that every person should do					
13. I am hesitant to give blood because I cannot be sure that the blood I give reaches those in need					
14. I believe that the organization I gave blood to did not deliver the blood to those in need					
15. The length of the pre-donation questionnaire causes me to give up giving blood					
16. Waiting before giving blood makes me give up					
17. Before giving blood, the physical discomfort of another donor before me (inability to find a vein, excessive bleeding...) affects my giving blood					
18. I do not donate blood for fear of physical decline before donation					
19. Hygienic staff, venue, and medical equipment affect my decision to give blood					
20. The manual dexterity of the nurse who will take blood affects my next blood donation					
21. I give blood because it is a good deed					
22. I donate blood for someone’s blessing.					
23. Men should donate more blood because they are stronger than women					
24. If I do not have health insurance, I donate blood to understand my health status and for a blood test					
